# GWAS of habitual coffee consumption reveals a sex difference in the genetic effect of the 12q24 locus in the Japanese population

**DOI:** 10.1186/s12863-019-0763-7

**Published:** 2019-07-26

**Authors:** Huijuan Jia, Shun Nogawa, Kaoru Kawafune, Tsuyoshi Hachiya, Shoko Takahashi, Maki Igarashi, Kenji Saito, Hisanori Kato

**Affiliations:** 1 Health Nutrition, Department of Applied Biological Chemistry, Graduate School of Agricultural and Life Sciences, The University of Tokyo, 1-1-1 Yayoi, Bunkyo-ku, Tokyo, 113-8657 Japan; 2 Genequest Inc., 5-29-11 Siba, Minato-ku, Tokyo, 108-0014 Japan; 3 Genome Analytics Japan Inc., 15-1-3205, Tomihisa-cho, Shinjuku-ku, Tokyo, 162-0067 Japan; 4 Department of Molecular Endocrinology, National Research Institute for Child Health and Development, 2-10-1 Okura, Setagaya-ku, Tokyo, 157-8535 Japan

**Keywords:** 12q24 locus, AHR, Coffee consumption, East Asians, Genome-wide association study

## Abstract

**Background:**

Studies on genetic effects of coffee consumption are scarce for Asian populations. We conducted a genome-wide association study (GWAS) of habitual coffee consumption in Japan using a self-reporting online survey.

**Results:**

Candidate genetic loci associated with habitual coffee consumption were searched within a discovery cohort (*N* = 6,264) and confirmed in a replication cohort (*N* = 5,975). Two loci achieved genome-wide significance (*P* < 5 × 10^− 8^) in a meta-analysis of the discovery and replication cohorts: an Asian population-specific 12q24 (rs79105258; *P* = 9.5 × 10^− 15^), which harbors *CUX2*, and 7p21 (rs10252701; *P* = 1.0 × 10^− 14^), in the upstream region of the aryl hydrocarbon receptor (*AHR*) gene, involved in caffeine metabolism. Subgroup analysis revealed a stronger genetic effect of the 12q24 locus in males (*P* for interaction = 8.2 × 10^− 5^). Further, rs79105258 at the 12q24 locus exerted pleiotropic effects on body mass index (*P* = 3.5 × 10^− 4^) and serum triglyceride levels (*P* = 8.7 × 10^− 3^).

**Conclusions:**

Our results consolidate the association of habitual coffee consumption with the 12q24 and 7p21 loci. The different effects of the 12q24 locus between males and females are a novel finding that improves our understanding of genetic influences on habitual coffee consumption.

**Electronic supplementary material:**

The online version of this article (10.1186/s12863-019-0763-7) contains supplementary material, which is available to authorized users.

## Background

Many epidemiologic studies have investigated the health benefits of drinking coffee, which is one of the most popular beverages globally. These benefits include a reduced risk of type 2 diabetes mellitus [1, 2], cardiovascular disease [3, 4], Parkinson’s and Alzheimer’s diseases [5, 6], and colorectal and liver cancers [7–9]. Conversely, coffee consumption is not recommended for pregnant women [10] or individuals with sleep disorders [11, 12].

Recently, genome-wide association studies (GWASs) have revealed genetic variants associated with the habitual intake of coffee [13, 14]. Studies conducted in European and American populations have identified common variants at the cytochrome P450 1A1 and 1A2 (*CYP1A1* and *CYP1A2*) loci on chromosome 15 and in a region close to the aryl-hydrocarbon receptor (*AHR*) gene on chromosome 7, as well as several novel genetic variants [15–20]. CYP1A2 is the primary enzyme that metabolizes caffeine, while *AHR* plays a regulatory role in inducing CYP1A1 and CYP1A2 expression [15–17].

Genetic studies investigating the association between habitual coffee/caffeine intake and genetic factors in Asian populations are scarce. To our knowledge, only one GWAS on habitual coffee consumption in East Asian populations has been reported: a GWAS conducted on a Japanese population, which found that the 12q24.12–13 locus is strongly associated with habitual coffee consumption [21].

In this study, we conducted a GWAS of the Japanese population to identify genetic loci associated with coffee-drinking habits. We confirmed the association between habitual coffee consumption and the loci 12q24 and 7p21; the latter was previously identified in populations of European decent in the United States [18]. Subsequently, we performed a subgroup analysis stratified by sex and age to further explore the link between the 12q24 and 7p21 loci and coffee consumption.

## Methods

### Study design

The study participants were customers of the Japanese DTC genetic testing service, HealthData Lab, which is provided by GeneQuest Inc. (Tokyo, Japan) and Yahoo! Japan Corporation (Tokyo, Japan). All participants were more than 18 years old, had answered an online self-reported survey, and had consented to the use of their genotype and questionnaire data for this study. Written informed consent was obtained. The study purpose was explained to the participants and a further agreement was obtained, allowing participants to opt-out. Among 12,621 participants, one opted out of participation in this study was excluded.

The discovery cohort consisted of study participants who lived in the eastern regions of Japan (Hokkaido, Tohoku, and Kanto-Koshinetsu) at the time of the online survey, and the replication cohort consisted of subjects who lived in western regions of Japan (Tokai-Hokuriku, Kinki, Chugoku-Shikoku, Kyushu, and Okinawa). Principal component analysis (PCA) in a previous study showed that the genetic distribution of the eastern region partially overlapped with that of the western region [22]. In a previous GWAS of coffee consumption using Japanese populations, almost all the subjects lived in western regions of Japan [21]. We used the discovery cohort to perform a genome-wide search for candidate loci associated with habitual coffee consumption. Loci that met suggestive significance (*P* < 1 × 10^− 5^) were further examined in the replication cohort. In the meta-analysis of the discovery and replication cohorts, loci that achieved genome-wide significance (*P* < 5 × 10^− 8^) were considered to be associated with habitual coffee consumption.

### Phenotype measurement

Habitual coffee consumption was assessed using an online survey that asked participants the following questions: “How many cups of coffee (instant or regular) do you drink?” and “How many cups of coffee (can, PET bottles, or paper pack) do you drink?”, with a choice of seven answers for each: (i) hardly drink, (ii) less than or equal to two cups per week, (iii) from three to four cups per week, (iv) from five to six cups per week, (v) from one to two cups per day, (vi) from three to four cups per day, and (vii) more than or equal to five cups per day. These categories were converted into continuous variables: (i) 0.0 cups per day, (ii) 0.29 (= 2/7) cups per day, (iii) 0.5 (= 3.5/7) cups per day, (iv) 0.79 (= 5.5/7) cups per day, (v) 1.5 cups per day, (vi) 3.5 cups per day, and (vii) 5.0 cups per day. The sum of the two continuous values calculated for each question was used as the habitual coffee consumption.

In addition, the participants were asked: “Have you undergone a health check-up within the past year?” If the participants responded positively, they were then asked: “Please provide your height, weight, total cholesterol (TC), triglyceride (TG), and hemoglobin A1c (HbA1c) levels.” Participant body-mass index (BMI) was calculated by dividing weight (kg) by the square of height (m). Because TC, TG, and HbA1c values were obtained as free text rather than as structured predefined categories, data points with extreme values (< 5th percentile or > 95th percentile) were excluded for each variable. Females were asked “Did you experience menopause? If yes, at what age did you experience menopause?”. Based on the result of this question, we divided females into two subgroups, before and after menopause.

### DNA sampling and genotyping assay

An Oragene®•DNA (OG-500) Collection Kit (DNA Genotek, Ottawa, Ontario, Canada) was used for the collection, stabilization, and transportation of saliva samples. The samples were genotyped using two platforms: the Illumina HumanCore-12+ Custom BeadChip (Illumina, San Diego, CA, USA), which contains 302,073 markers; and the Illumina HumanCore-24+ Custom BeadChip, which contains 309,725 markers. For analysis in the present study, we used 296,675 SNPs that were present in both genotyping platforms.

### Quality control of genotype data

At the individual level, we excluded subjects who lived in non-Japanese areas or whose residence area was unavailable (*N* = 17). Sex agreement between genotype and questionnaire data was checked by imputing sex from the X chromosome genotype data in PLINK [23], software version 1.90b3.42. We found that the imputed sex of 16 individuals in the discovery cohort and 8 individuals in the replication cohort was uncertain or not consistent with self-reported sex; those individuals were excluded from further analyses because of the potential unreliability of the questionnaire answers. Next, kinship was examined by pairwise identity-by-descent (IBD) estimation in PLINK, from which an additional 127 individuals (59 in the discovery cohort and 68 in the replication cohort) were excluded (PI_HAT > 0.1875). Genetic ancestry was estimated by a PCA method implemented in the Eigensoft [24, 25] program, version 6.1.3. Then, 146 subjects (77 in the discovery cohort and 69 in the replication cohort) whose ancestry was not included in the Japanese cluster were excluded. Furthermore, 67 subjects (39 in the discovery cohort and 26 in the replication cohort) were excluded because age and/or coffee consumption data were unavailable. All samples showed a call rate of ≥95%. At the SNP level, we excluded variants that were located on sex chromosomes, had a low call rate (< 95%), showed a discrepancy from the Hardy-Weinberg equilibrium (*P* < 1 × 10^− 6^), and had a low minor allele frequency (< 1%). This SNP-level quality control filtering resulted in 218,384 SNPs remaining for the discovery cohort.

### Genotype imputation

We performed genotype imputation for the discovery cohort using the 1000 Genomes Phase3 (version 5) reference panel [26]. Several previous GWASs in the Japanese population extracted East Asian subjects from the imputation panel and the ethnicity-matched reference panel was used for genotype imputation [27, 28]. Meanwhile, Roshyara et al., showed that, for the Japanese population, the accuracy of genotype imputation with the ethnicity-mixed reference panel is greater than that with the ethnicity-matched reference panel [29]. Thus, recent GWASs using the Japanese or East Asian populations have utilized the ethnicity-mixed reference panel [21, 30–33], including a previous GWAS of habitual coffee consumption in the Japanese population [21]. For these reasons, we used the ethnicity-mixed reference panel for imputation.

The 218,384 SNPs that passed our quality control criteria were pre-phased using EAGLE2 (version 2.4) software [34]. Post-phase imputation was executed using Minimac3 (version 2.0.1) software [35]. We excluded variants that had a low imputation quality (*R*^2^ <  0.8) and a low minor allele frequency (< 1%). As a result, dosage data for 5,264,155 variants were used for discovery analysis.

### Genome-wide association study (GWAS)

We used a linear regression method to test for association between habitual coffee consumption and dosage estimate for each variant, assuming an additive model. The covariates included age and sex. The estimated regression coefficient for each SNP (denoted by β) and standard error (SE) were calculated for the minor alleles. The genome-wide tests were performed using PLINK. Regression coefficients from the discovery and replication cohorts were converted to regression coefficients and *P*-values for the meta-analysis using a fixed-effect model and the inverse-variance weighting method with METAL software (version 2011-03-25) [36].

Manhattan and quantile-quantile plots were created using the R software package qqman [37] version 0.1.4. For SNPs that reached significance, we created regional-association plots using LocusZoom [38] version 1.3. In these plots, we used linkage disequilibrium (LD) information from the 1,000 Genomes Project 2014 East Asian (ASN) database on the hg19 genome build to estimate recombination rates.

### Statistical analysis

Continuous dosage data for 12q24 and 7p21 lead variants (rs79105258 and rs10252701, respectively) were converted to discretized genotype data (coded as 0, 1, or 2) by assigning the genotype that had the highest posterior probability estimated in the genotype imputation process as described previously [39]. In subgroup analyses stratified by sex or age, the associations between polymorphisms and habitual coffee consumption were tested using multivariate regression under an additive genetic model for each subgroup. The regression model used the level of coffee consumption as an objective variable and the number of alternative alleles as explanatory variable. Age and cohort region (eastern or western regions in Japan) were adjusted for the sex-stratified subgroup analysis. Sex and cohort regions were also adjusted for the age-stratified subgroup analysis. The heterogeneity of genetic effects was tested by adding a multiplicative interaction term to the relevant multivariate regression model.

In the analysis of pleiotropic effects, the associations between rs79105258 and BMI, TC, TG, and HbA_1c_ were tested using linear regression under an additive genetic model, which used a clinical phenotype (BMI, TC, TG or HbA_1c_) as an objective variable and the number of alternative alleles as an explanatory variable. Age, sex, and cohort region were adjusted for this analysis.

## Results

### Characteristics of study subjects

We performed a GWAS of coffee consumption using data collected by the Japanese direct-to-consumer (DTC) genetic testing service HealthData Lab. After quality control, a total of 218,384 SNPs and 6,264 individuals remained for GWAS discovery and 5,975 subjects for subsequent replication analysis. Characteristics of the discovery and replication cohorts are shown in Table 1. The proportion of females in the discovery cohort (45.8%) was slightly smaller than that in the replication cohort (47.7%). Mean age was slightly lower in the discovery cohort (49.9 ± 13.0 years old) than in the replication cohort (50.6 ± 13.3) and BMI in the discovery cohort (23.1 ± 3.8) was slightly greater than that in the replication cohort (23.0 ± 3.6). In the discovery population, the proportion of current alcohol drinkers and the level of alcohol consumption were significantly higher (*P* <  0.05) than those in the replication population (Table 1). The average coffee consumption was 2.01 ± 1.43 and 2.04 ± 1.41 cups/day for the discovery and replication cohorts, respectively. No significant difference in coffee consumption was observed between the two cohorts (*P* = 0.30).

**Table 1:** Characteristics of study subjects

	Discovery (East Japan)	Replication (West Japan)	*P*
*N*	6,264	5,975	–
Female, %	45.8	47.7	0.04
Age, years (mean ± SD)	49.9 ± 13.0	50.6 ± 13.3	0.005
BMI, kg/m^2^ (mean ± SD)	23.1 ± 3.8	23.0 ± 3.6	0.03
Coffee consumption, cups/day (mean ± SD)	2.01 ± 1.43	2.04 ± 1.41	0.30
Current alcohol drinkers, %	63.9	59.3	< 0.001
Current alcohol consumption, g/day (mean ± SD)^a^	12.2 ± 14.2	10.9 ± 12.6	< 0.001

^a^Among current alcohol drinkers, *BMI* Body mass index, *SD* Standard deviation

*P*-values were calculated using Fisher’s exact test for sex and current alcohol drinkers, and Student’s *t*-test for all other variables

### Discovery GWAS, replication, and meta-analysis

**Fig. 1:**
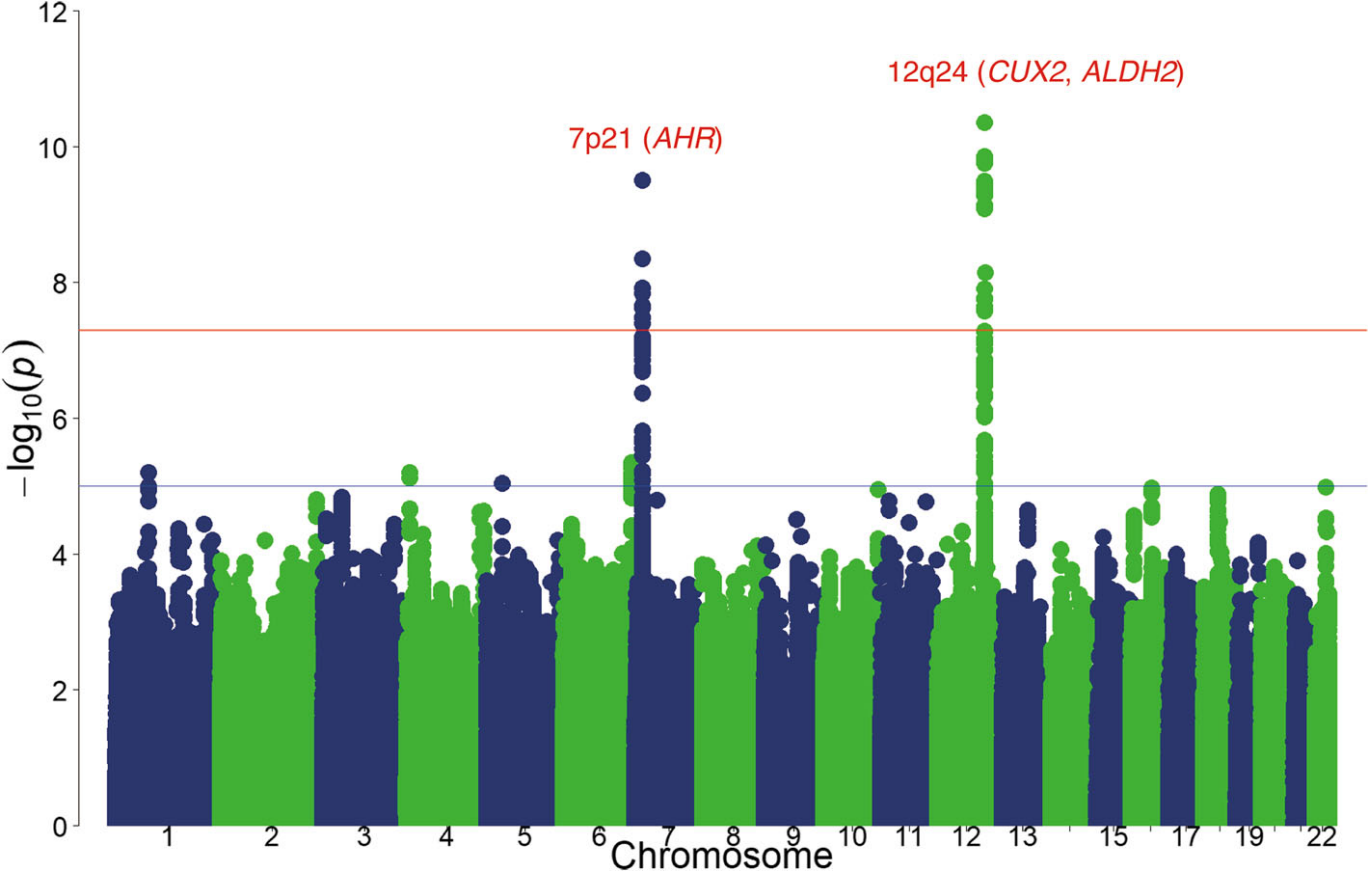
Manhattan plot for the GWAS of habitual coffee consumption. SNPs are organized by chromosome and position along the *x*-axis. The *y*-axis represents the negative logarithm of the association of each SNP with coffee consumption, with the red line and blue line corresponding to *P* < 5.0 × 10^− 8^ and *P* < 1.0 × 10^− 5^, respectively. Regions with significant association are labeled in red

In the discovery GWAS, the association of habitual coffee consumption with all variants that passed quality control filters (5,264,155 variants, including 218,384 directly genotyped SNPs) was tested, with adjustment for age and sex (Fig. 1). We did not use principal components as adjustment variables because the genomic inflation factor was close to 1.0 (λ_GC_ = 1.009; 95% confidence interval [CI], 1.007–1.011) in the genome-wide scan (Additional file 2: Figure S1). The discovery analysis identified six loci that met suggestive significance (*P* < 1 × 10^− 5^) (Table 2).

**Table 2:** Variants associated with habitual coffee consumption

Variant	Chr	Position	Gene	EA	NEA	Population	EAF	Beta	SE (Beta)	*P*_association_	*I* ^2^	*P*_heterogeneity_
rs518341	1	78,896,492	*GIPC2– PTGFR*	G	A	Discovery	0.315	0.133	0.030	6.4 × 10^− 6^	–	–
						Replication	0.317	0.009	0.030	0.77	–	–
						Meta-analysis	0.316	0.072	0.021	5.9 × 10^−4^	88.6	3.1 × 10^−3^
rs11722777	4	7,590,819	*SORCS2*	A	G	Discovery	0.816	−0.167	0.037	6.4 × 10^−6^	–	–
						Replication	0.821	−0.075	0.038	0.05	–	–
						Meta-analysis	0.819	−0.123	0.027	3.9 × 10^−6^	66.3	0.08
rs10941308	5	36641,007	*SLC1A3*	A	G	Discovery	0.115	0.189	0.043	9.3 × 10^−6^	–	–
						Replication	0.118	−0.026	0.044	0.55	–	–
						Meta-analysis	0.117	0.084	0.031	5.8 × 10^−3^	92.0	4.2 × 10^−4^
rs10648495	6	162,128,756	*PRKN*	CTCTTT	C	Discovery	0.463	−0.130	0.028	4.5 × 10^−6^	–	–
						Replication	0.459	0.040	0.029	0.16	–	–
						Meta-analysis	0.461	−0.046	0.020	0.02	94.4	2.4 × 10^−5^
rs10252701	7	17,280,513	*AHR*	C	A	Discovery	0.322	−0.194	0.031	3.2 × 10^−10^	–	–
						Replication	0.308	−0.146	0.032	4.0 × 10^−6^	–	–
						Meta-analysis	0.315	−0.171	0.022	1.0 × 10^−14^	15.1	0.28
rs79105258	12	111,718,231	*CUX2*	A	C	Discovery	0.268	0.211	0.032	4.4 × 10^−11^	–	–
						Replication	0.298	0.137	0.031	1.3 × 10^−5^	–	–
						Meta-analysis	0.283	0.173	0.022	9.5 × 10^−15^	63.2	0.10

*Chr* Chromosome number, *EA* Effect allele, *NEA* Non-effect allele, *EAF* Effect allele frequency, *SE* Standard error

These six candidate loci were further examined in the replication cohort. Three loci (1p31, 5p13 and 6q26) were not replicated. The remaining three (4p16, 7p21 and 12q24) were significantly associated with habitual coffee consumption (*P* <  0.05). After meta-analysis of the discovery and replication cohorts, two of these loci (7p21 and 12q24) achieved genome-wide significance (*P* < 5 × 10^− 8^). Heterogeneity in genetic effects between the discovery and replication cohorts was not significant for the 7p21 and 12q24 loci (Table 2).

Additional file 2: Figure S2 shows genomic regional plots for the two loci associated with habitual coffee consumption. The strongest association was found for the locus 12q24 (lead variant, rs79105258; *P* for meta-analysis = 9.5 × 10^− 15^), which harbors several genes, including aldehyde dehydrogenase 2 (*ALDH2*) and cut-Like homeobox 2 (*CUX2*). The second strongest association was found for 7p21 (lead variant, rs10252701; *P* for meta-analysis = 1.0 × 10^− 14^), an upstream region of the aryl hydrocarbon receptor (*AHR*) gene.

For sensitivity analysis, we categorized subjects according to habitual coffee consumption: (i) no coffee (i.e., 0.00 cups per day), (ii) 0.00 to 1.00 cups of coffee per day, (iii) 1.00 to 3.00 cups of coffee per day, and (iv) more than 3.00 cups of coffee per day. The cut-off values were determined according to previous studies [40–42]. Next, we investigated the association of the categorized variable with the 7p21 and 12q24 variants. In this analysis, both the discovery and replication cohorts were pooled. The results showed that the frequency of the rs79105258 A allele (at the 12q24 locus) was significantly higher in the group of subjects who drank more than 3.00 cups of coffee per day than in those who did not drink coffee (odds ratio [OR] per allele, 1.39; *P* = 1.4 × 10^− 10^) (Table 3). The frequency of the rs10252701 C allele at the 7p21 locus was decreased in the group of the subjects who drank more than 3.00 cups of coffee per day compared with that in those who did not drink coffee (OR, 0.76; *P* = 9.1 × 10^− 9^). These results confirmed the association between habitual coffee consumption and the 12q24 and 7p21 loci.

**Table 3:** Genotype and allele frequencies for rs10252701 (*AHR*) and rs79105258 (*CUX2*) according to habitual coffee consumption level

SNP	Coffee consumption (Cups/day)				
	Allele	0.00	0.00–1.00	1.00–3.00	3.00–10.00
rs10252701	AA [N (%)]	740 (43.7)	1151 (43.4)	2481 (46.6)	1339 (52.2)
*N* = 12,239	CA [N (%)]	753 (44.5)	1204 (45.4)	2311 (43.4)	1010 (39.4)
	CC [N (%)]	200 (11.8)	299 (11.3)	535 (10.0)	216 (8.4)
	OR (95% CI)	1 (ref.)	1.00 (0.91–1.09)	0.90 (0.83–0.98)	0.76 (0.69–0.84)
	*P*	–	0.92	0.01	9.1 × 10^−9^
rs79105258	CC [N (%)]	1015 (60.0)	1594 (60.1)	2960 (55.6)	1269 (49.5)
*N* = 12,239	AC [N (%)]	578 (34.1)	872 (32.9)	2040 (38.3)	1089 (42.5)
	AA [N (%)]	100 (5.9)	188 (7.1)	327 (6.1)	207 (8.1)
	OR (95% CI)	1 (ref.)	1.03 (0.93–1.14)	1.13 (1.04–1.24)	1.39 (1.26–1.54)
	*P*	–	0.58	6.7 × 10^−3^	1.4 × 10^−10^

*OR* Odds ratio, *CI* Confidence interval. All ORs and *P*-values were calculated by a logistic regression method with the additive genetic model

We sought to find additional signals by genome-wide comparisons of heavy coffee consumers (≥ 3.00 cups of coffee per day) with others (< 3.00 cups of coffee per day). As a result, 12q24 and 7p21 loci achieved genome-wide significance, although no additional loci were significantly associated with coffee consumption (*P* > 5 × 10^− 8^) (Additional file 1: Table S1). In addition, we conducted genome-wide analysis with dominant and recessive models, identifying no additional significant loci (Additional file 1: Tables S2 and S3).

### Potential confounding factors

To examine whether the association of habitual coffee consumption with 12q24 and 7p21 variants may be mediated by potential confounding factors, including BMI, alcohol consumption, alcohol drinking frequency, and smoking status, we performed association tests with additional adjustment for those potential confounding variables (Additional file 1: Table S4). The effects of the 12q24 and 7p21 variants were not attenuated by the addition of any potential confounding variables, suggesting that the associations between habitual coffee consumption and the 12q24/7p21 variants are independent of BMI, alcohol consumption, and smoking.

### Subgroup analysis according to sex and age

To determine whether the genetic effects of variants at the 12q24 and 7p21 loci are different between males and females or between younger and older participants, the associations were tested for each subgroup defined according to sex or age. In this analysis, subjects from the discovery and replication cohorts were pooled. Subjects younger than the median age (51 years old) were considered the younger subgroup and the remaining subjects were categorized as the older subgroup.

**Fig. 2:**
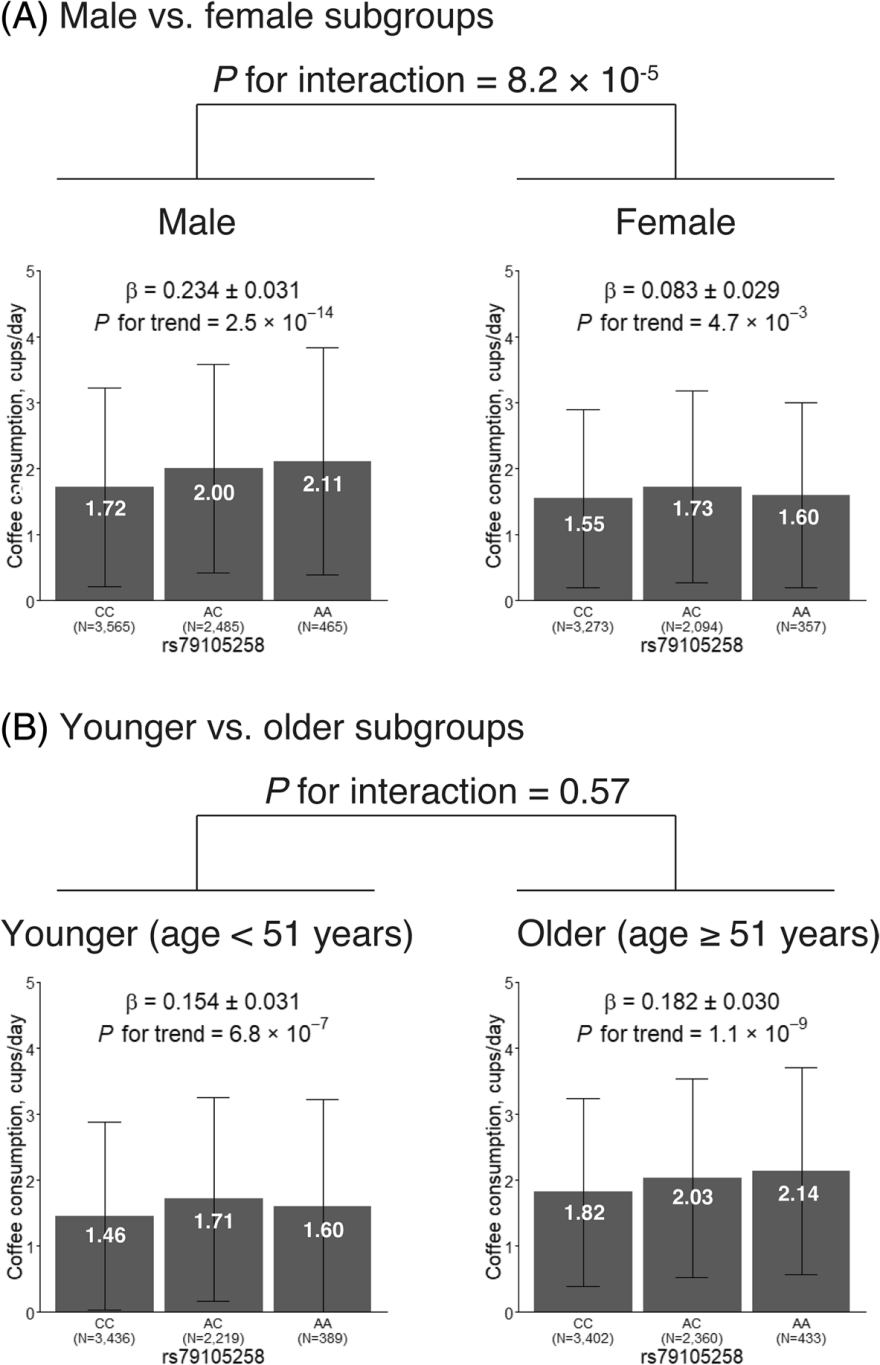
Effects of the 12q24 locus variant, rs79105258, on habitual coffee consumption stratified by sex and age. **a** Male vs. female subgroups, **b** Younger vs. older subgroups. The coffee consumption level (white lettering) is marked for each allele, GG, AG, and AA for rs79105258. Error bars indicate standard deviation. β denotes the estimated regression coefficient

The effect size (i.e., mean difference in habitual coffee consumption per allele) of the rs79105258 variant (at the 12q24 locus) was 0.254 (standard error [SE], 0.031) cups per day in males and 0.083 (SE, 0.029) in females (Fig. 2a). This sex difference was significant (*P* for interaction = 8.2 × 10^− 5^), suggesting that the genetic effect of the 12q24 locus is stronger in males than in females. The effect size in the younger subgroup (0.154; SE, 0.031) was not different from that in the older subgroup (0.182; SE, 0.030) (*P* for interaction = 0.57; Fig. 2b).

For the rs10252701 variant at the 7p21 locus, no significant differences in genetic effects were found between males and females (*P* for interaction = 0.13; Additional file 2: Figure S3A), or between the younger and older subgroups (*P* for interaction = 0.45; Additional file 2: Figure S3B).

### Pleiotropic genetic effects of the 12q24 locus on BMI and TG levels

We examined the pleiotropic effects of the 12q24 and 7p21 variants on BMI, TC, TG, and HbA_1c_. The rs79105258 A allele was associated with decreased BMI (*P* = 3.5 × 10^− 4^) and TG (*P* = 8.7 × 10^− 3^) after adjustment for age, sex, and cohort region (Table 4). Similar results were found after further adjustment for alcohol consumption (Additional file 1: Tables S5 and S6).

**Table 4:** Pleiotropic effects of coffee-associated variants

	rs10252701			rs79105258		
	Beta	SE (Beta)	*P*	Beta	SE (Beta)	*P*
BMI	0.021	0.049	0.67	−0.188	0.053	3.5 × 10^−4^
Total cholesterol	−0.690	0.635	0.28	0.120	0.674	0.86
Triglyceride	−0.186	0.888	0.83	−2.488	0.948	8.7 × 10^−3^
HbA1c	0.015	0.011	0.17	−0.004	0.012	0.71

*BMI* Body mass index, *SE* Standard error. Adjusted for age, sex, and cohort region

## Discussion

We conducted a GWAS of habitual coffee consumption in the Japanese population, identifying two loci, 12q24 and 7p21, that achieved genome-wide significance. Both loci have been previously linked to coffee consumption [15–20], and our study further verifies this association. In subsequent analyses, we examined the differences in genetic effects of the two loci and found that the effect of the 12q24 locus was much stronger in males than in females. To our knowledge, this finding has not been reported previously.

In a previous meta-analysis of a Japanese population [21], 24 novel SNPs in the 12q24.12–13 region, which spans 13 genes, showed genome-wide significance with habitual coffee consumption. Because these genes are in strong linkage disequilibrium, the authors suggested that the 12q24.12–13 region is likely responsible for variations in coffee consumption, but could not conclude which gene located in this region is responsible for influencing coffee consumption. It is well known that the 12q24 locus has pleiotropic effects on alcohol consumption [43], BMI [44], TG [45], the risk of coronary heart disease [46], and esophageal cancer [47]. The pleiotropic effects on BMI and TG were confirmed in our datasets.

The identification of a common SNP, rs10252701, near the *AHR* gene, is consistent with GWASs conducted in several European and American populations [15–20]. The *AHR* gene is involved in caffeine metabolism and encodes a ligand-activated transcription factor that is an upstream inducer of *CYP1A1* and *CYP1A2* expression*.* We found decreased consumption of coffee in individuals carrying the C allele for *AHR* rs10252701.

The rs79105258 C allele (at the 12q24 locus) was significantly associated with higher habitual coffee consumption after adjustment for age, sex, and cohort region. As the 12q24 locus harbors the *ALDH2* gene, which encodes an enzyme involved in alcohol metabolism, we investigated whether the association between 12q24 and coffee consumption is influenced by alcohol consumption or the association is independent of alcohol consumption. As the result, we confirmed that the association of the 12q24 locus with coffee consumption is independent of alcohol consumption, consistent with findings from a previous Japanese population GWAS [21].

Our study found a remarkable sex difference in the genetic effect of the 12q24 locus on coffee consumption. This difference might be related to sex steroid hormones. To examine whether the sex difference could be explained by sex hormones, we compared the genetic effect of the 12q24 locus on habitual coffee consumption between females before and after menopause (Additional file 2: Figure S4). The result showed that the genetic effect in females after menopause (β = 0.086; SE = 0.043; *P* = 4.4 × 10^− 2^) was not significantly different from that before menopause (β = 0.082; SE = 0.040; *P* = 3.9 × 10^− 2^) (*P* for interaction = 0.85). Further large-scale genetic studies are needed to reveal whether sex steroids play any role in the sex difference of the genetic effects of the 12q24 locus.

To date, several Mendelian randomization (MR) studies have investigated the causal role of coffee or caffeine use on health outcomes, including risks for type 2 diabetes, cardiovascular disease, and cancers [48]. These MR studies have provided no consistent support for a causal role of coffee or caffeine on health outcomes, possibly due to low statistical power, potential pleiotropy, and/or risk of collider bias [48]. Our association of the 12q24 and 7p21 with habitual coffee consumption in the Japanese population might be useful for future MR studies that investigate the preventive effect of coffee drinking, particularly for East Asian populations.

It is important to highlight the methodological limitations of this study. Details regarding cup size, coffee type (e.g., decaffeinated, boiled, or filtered), and precise chemical composition (caffeine content) were not available. As discussed in a previous study [13], habitual coffee consumption data were not normally distributed in our population. Additionally, phenotypic variables (e.g., BMI and TG) were obtained from a self-reported survey and may not be entirely accurate. For population-specific variants, the accuracy of genotype imputation using the ethnicity-mixed reference panel might be less accurate compared to that using the ethnicity-matched reference panel. Despite these limitations, our results were consistent with those of previous studies [15–21].

## Conclusions

In conclusion, this study consolidates the association of habitual coffee consumption with the 12q24 and 7p21 loci. We have for the first time revealed that the genetic effect of the 12q24 locus is stronger in males than in females. Our present findings contribute to our understanding of the genetic factors and sex differences influencing coffee consumption, which has implications for lifestyle guidance based on genetic data for East Asian populations.

## Additional files

Additional file 1: **Table S1.** Results of genome-wide association analysis that compares heavy coffee consumers with others. **Table S2.** Results of genome-wide association analysis based on a dominant model. **Table S3.** Results of genome-wide association analysis based on a recessive model. **Table S4.** Adjustment for potential confounding factors. **Table S5.** Pleiotropic effects with adjustment for age, sex, cohort region, and alcohol consumption. **Table S6.** Pleiotropic effects with adjustment for age, sex, cohort region, and alcohol frequency. (PPTX 65 kb)

Additional file 2: **Figure S1.** Quantile-quantile plot for the genome-wide analysis of coffee consumption. **Figure S2.** The genomic regional plot from the association analysis of coffee consumption. **Figure S3.** Effects of a 7p21 variant on habitual coffee consumption stratified by sex and age. **Figure S4.** Genetic effect of the 12q24 locus in females before and after menopause. (PPTX 748 kb)

## Data Availability

The data generated or analyzed in this study are included in this published article and its supplementary information files. Other data are available from the authors on reasonable request.

## Abbreviations

*AHR*: Aryl-hydrocarbon receptor
ALDH2: Aldehyde dehydrogenase 2
BMI: Body mass index
CUX2: Cut like homeobox 2
DTC: Direct-to-consumer
GWAS: Genome-wide association studies
HbA1c: Haemoglobin A1c
OR: Odds ratio
TC: Total cholesterol
TG: Triglyceride
