# Supplementary material for: GWAS of habitual coffee consumption reveals a sex difference in the genetic effect of the 12q24 locus in the Japanese population
Source: BMC Genet. 2019 Jul 26;20:61. doi: 10.1186/s12863-019-0763-7 (PMC6659273; doi:10.1186/s12863-019-0763-7)
Supplement: Supplementary file 1 — Table S1. Results of genome-wide association analysis that compares heavy coffee consumers with others. Table S2. Results of genome-wide association analysis based on a dominant model. Table S3. Results of genome-wide association analysis based on a recessive model. Table S4. Adjustment for potential confounding factors. Table S5. Pleiotropic effects with adjustment for age, sex, cohort region, and alcohol consumption. Table S6. Pleiotropic effects with adjustment for age, sex, cohort region, and alcohol frequency. (PPTX 65 kb) [file 12863_2019_763_MOESM1_ESM.pptx]

## Slide 1
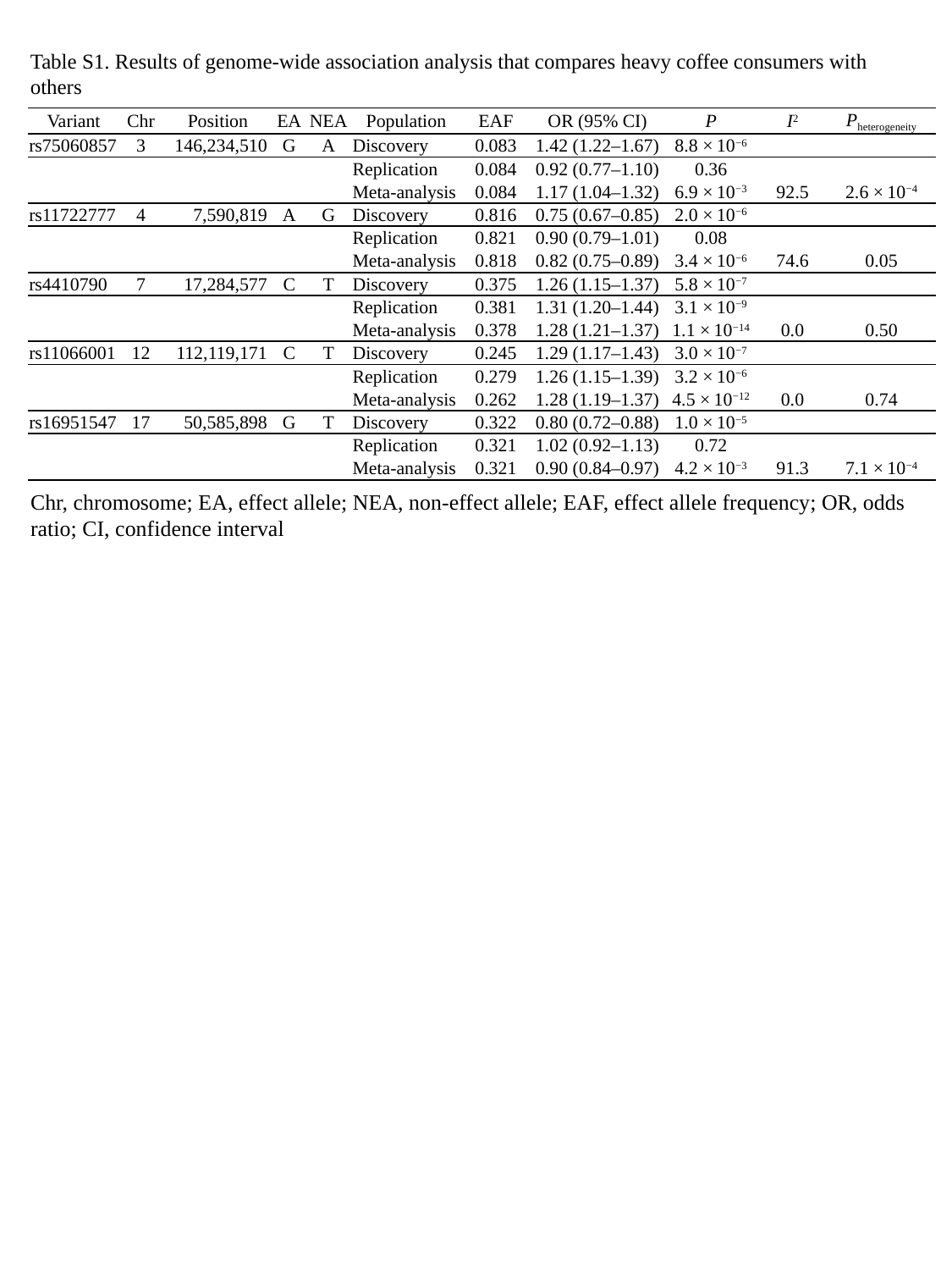

Table S1. Results of genome-wide association analysis that compares heavy coffee consumers with others
| Variant | Chr | Position | EA | NEA | Population | EAF | OR (95% CI) | P | I2 | Pheterogeneity |
| --- | --- | --- | --- | --- | --- | --- | --- | --- | --- | --- |
| rs75060857 | 3 | 146,234,510 | G | A | Discovery | 0.083 | 1.42 (1.22–1.67) | 8.8 × 10^−6^ | | |
| | | | | | Replication | 0.084 | 0.92 (0.77–1.10) | 0.36 | | |
| | | | | | Meta-analysis | 0.084 | 1.17 (1.04–1.32) | 6.9 × 10^−3^ | 92.5 | 2.6 × 10^−4^ |
| rs11722777 | 4 | 7,590,819 | A | G | Discovery | 0.816 | 0.75 (0.67–0.85) | 2.0 × 10^−6^ | | |
| | | | | | Replication | 0.821 | 0.90 (0.79–1.01) | 0.08 | | |
| | | | | | Meta-analysis | 0.818 | 0.82 (0.75–0.89) | 3.4 × 10^−6^ | 74.6 | 0.05 |
| rs4410790 | 7 | 17,284,577 | C | T | Discovery | 0.375 | 1.26 (1.15–1.37) | 5.8 × 10^−7^ | | |
| | | | | | Replication | 0.381 | 1.31 (1.20–1.44) | 3.1 × 10^−9^ | | |
| | | | | | Meta-analysis | 0.378 | 1.28 (1.21–1.37) | 1.1 × 10^−14^ | 0.0 | 0.50 |
| rs11066001 | 12 | 112,119,171 | C | T | Discovery | 0.245 | 1.29 (1.17–1.43) | 3.0 × 10^−7^ | | |
| | | | | | Replication | 0.279 | 1.26 (1.15–1.39) | 3.2 × 10^−6^ | | |
| | | | | | Meta-analysis | 0.262 | 1.28 (1.19–1.37) | 4.5 × 10^−12^ | 0.0 | 0.74 |
| rs16951547 | 17 | 50,585,898 | G | T | Discovery | 0.322 | 0.80 (0.72–0.88) | 1.0 × 10^−5^ | | |
| | | | | | Replication | 0.321 | 1.02 (0.92–1.13) | 0.72 | | |
| | | | | | Meta-analysis | 0.321 | 0.90 (0.84–0.97) | 4.2 × 10^−3^ | 91.3 | 7.1 × 10^−4^ |
Chr, chromosome; EA, effect allele; NEA, non-effect allele; EAF, effect allele frequency; OR, odds ratio; CI, confidence interval

## Slide 2
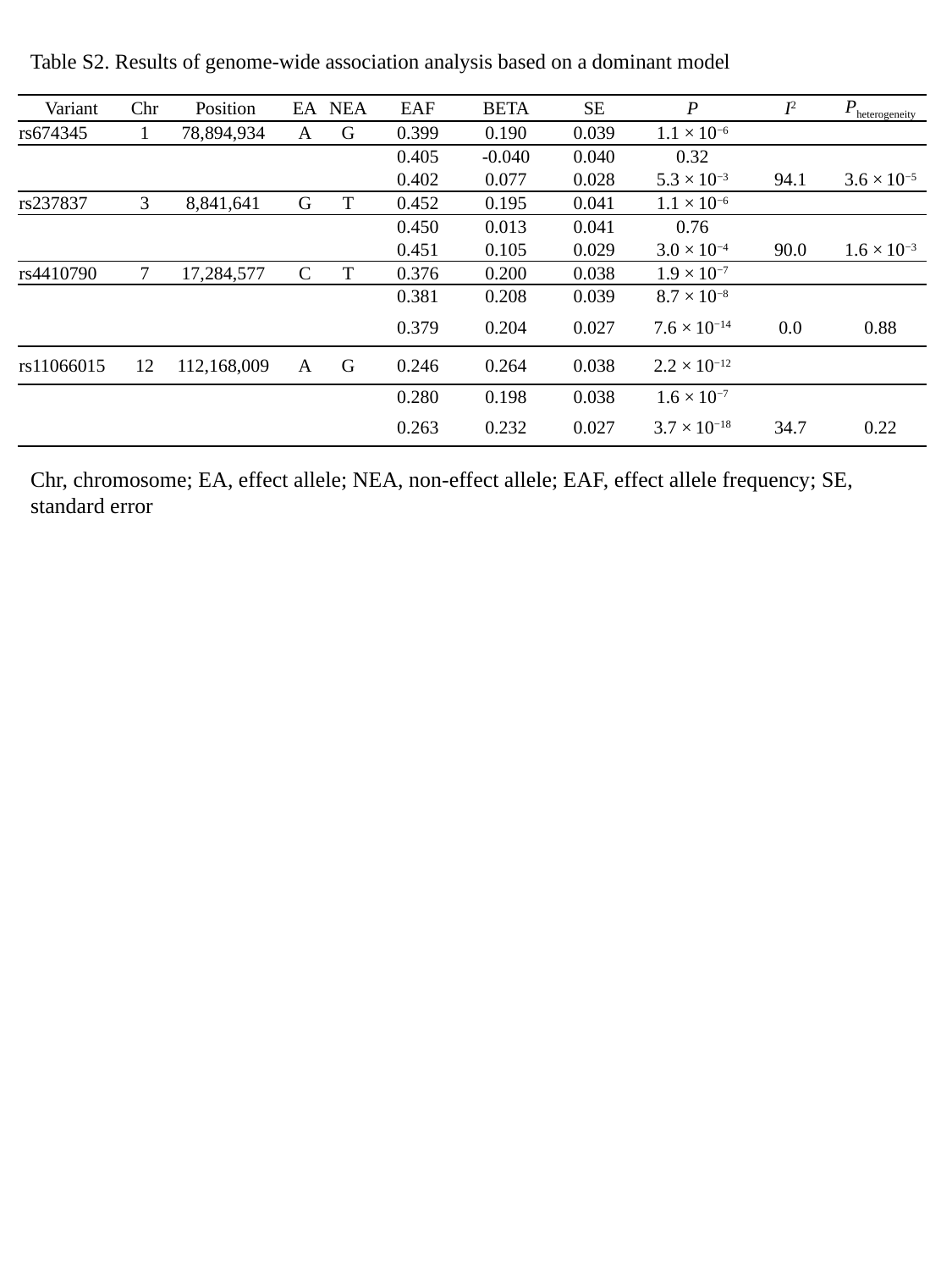

Table S2. Results of genome-wide association analysis based on a dominant model
| Variant | Chr | Position | EA | NEA | EAF | BETA | SE | P | I2 | Pheterogeneity |
| --- | --- | --- | --- | --- | --- | --- | --- | --- | --- | --- |
| rs674345 | 1 | 78,894,934 | A | G | 0.399 | 0.190 | 0.039 | 1.1 × 10^−6^ | | |
| | | | | | 0.405 | -0.040 | 0.040 | 0.32 | | |
| | | | | | 0.402 | 0.077 | 0.028 | 5.3 × 10^−3^ | 94.1 | 3.6 × 10^−5^ |
| rs237837 | 3 | 8,841,641 | G | T | 0.452 | 0.195 | 0.041 | 1.1 × 10^−6^ | | |
| | | | | | 0.450 | 0.013 | 0.041 | 0.76 | | |
| | | | | | 0.451 | 0.105 | 0.029 | 3.0 × 10^−4^ | 90.0 | 1.6 × 10^−3^ |
| rs4410790 | 7 | 17,284,577 | C | T | 0.376 | 0.200 | 0.038 | 1.9 × 10^−7^ | | |
| | | | | | 0.381 | 0.208 | 0.039 | 8.7 × 10^−8^ | | |
| | | | | | 0.379 | 0.204 | 0.027 | 7.6 × 10^−14^ | 0.0 | 0.88 |
| rs11066015 | 12 | 112,168,009 | A | G | 0.246 | 0.264 | 0.038 | 2.2 × 10^−12^ | | |
| | | | | | 0.280 | 0.198 | 0.038 | 1.6 × 10^−7^ | | |
| | | | | | 0.263 | 0.232 | 0.027 | 3.7 × 10^−18^ | 34.7 | 0.22 |
Chr, chromosome; EA, effect allele; NEA, non-effect allele; EAF, effect allele frequency; SE, standard error

## Slide 3
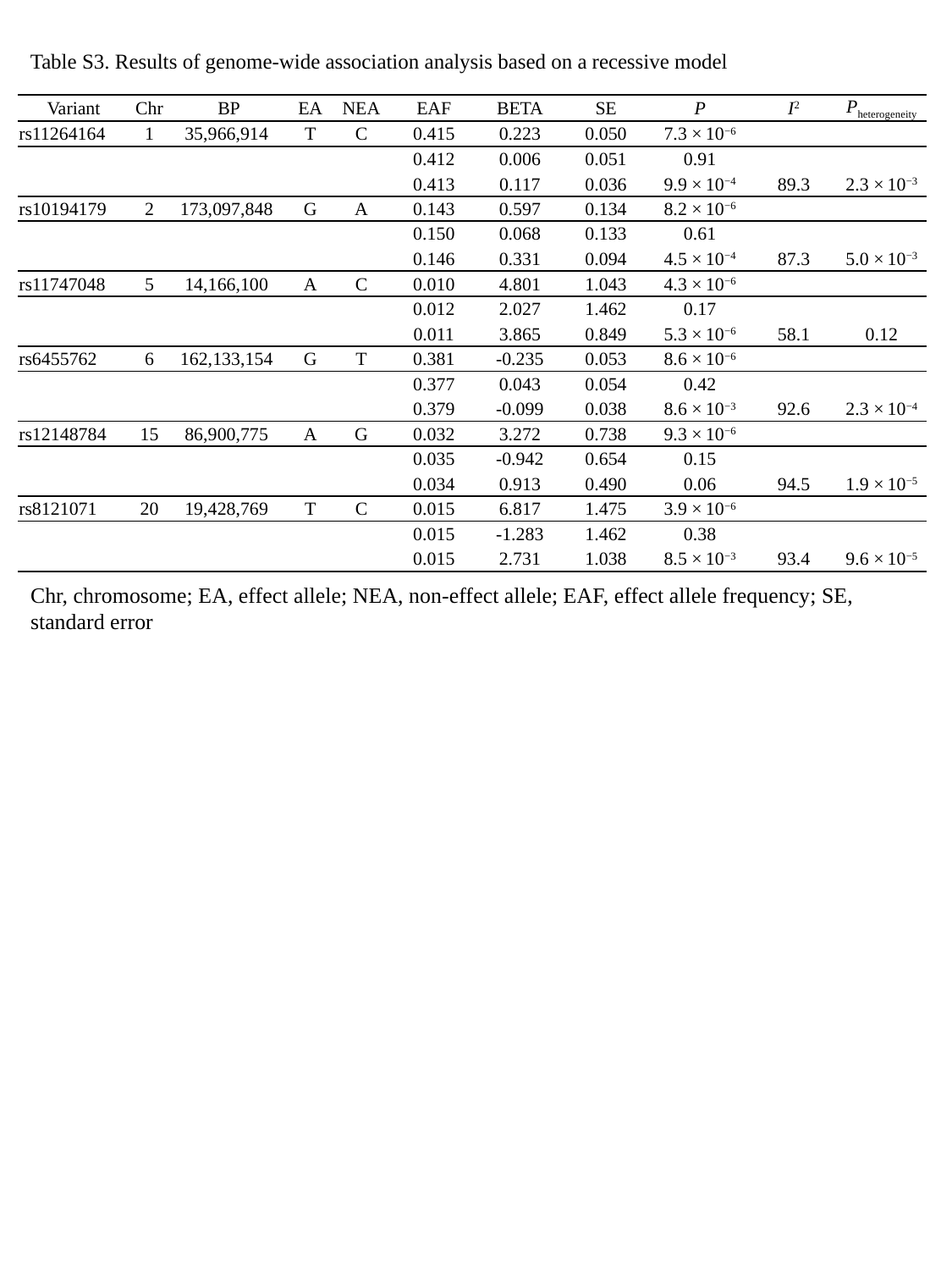

Table S3. Results of genome-wide association analysis based on a recessive model
| Variant | Chr | BP | EA | NEA | EAF | BETA | SE | P | I2 | Pheterogeneity |
| --- | --- | --- | --- | --- | --- | --- | --- | --- | --- | --- |
| rs11264164 | 1 | 35,966,914 | T | C | 0.415 | 0.223 | 0.050 | 7.3 × 10^−6^ | | |
| | | | | | 0.412 | 0.006 | 0.051 | 0.91 | | |
| | | | | | 0.413 | 0.117 | 0.036 | 9.9 × 10^−4^ | 89.3 | 2.3 × 10^−3^ |
| rs10194179 | 2 | 173,097,848 | G | A | 0.143 | 0.597 | 0.134 | 8.2 × 10^−6^ | | |
| | | | | | 0.150 | 0.068 | 0.133 | 0.61 | | |
| | | | | | 0.146 | 0.331 | 0.094 | 4.5 × 10^−4^ | 87.3 | 5.0 × 10^−3^ |
| rs11747048 | 5 | 14,166,100 | A | C | 0.010 | 4.801 | 1.043 | 4.3 × 10^−6^ | | |
| | | | | | 0.012 | 2.027 | 1.462 | 0.17 | | |
| | | | | | 0.011 | 3.865 | 0.849 | 5.3 × 10^−6^ | 58.1 | 0.12 |
| rs6455762 | 6 | 162,133,154 | G | T | 0.381 | -0.235 | 0.053 | 8.6 × 10^−6^ | | |
| | | | | | 0.377 | 0.043 | 0.054 | 0.42 | | |
| | | | | | 0.379 | -0.099 | 0.038 | 8.6 × 10^−3^ | 92.6 | 2.3 × 10^−4^ |
| rs12148784 | 15 | 86,900,775 | A | G | 0.032 | 3.272 | 0.738 | 9.3 × 10^−6^ | | |
| | | | | | 0.035 | -0.942 | 0.654 | 0.15 | | |
| | | | | | 0.034 | 0.913 | 0.490 | 0.06 | 94.5 | 1.9 × 10^−5^ |
| rs8121071 | 20 | 19,428,769 | T | C | 0.015 | 6.817 | 1.475 | 3.9 × 10^−6^ | | |
| | | | | | 0.015 | -1.283 | 1.462 | 0.38 | | |
| | | | | | 0.015 | 2.731 | 1.038 | 8.5 × 10^−3^ | 93.4 | 9.6 × 10^−5^ |
Chr, chromosome; EA, effect allele; NEA, non-effect allele; EAF, effect allele frequency; SE, standard error

## Slide 4
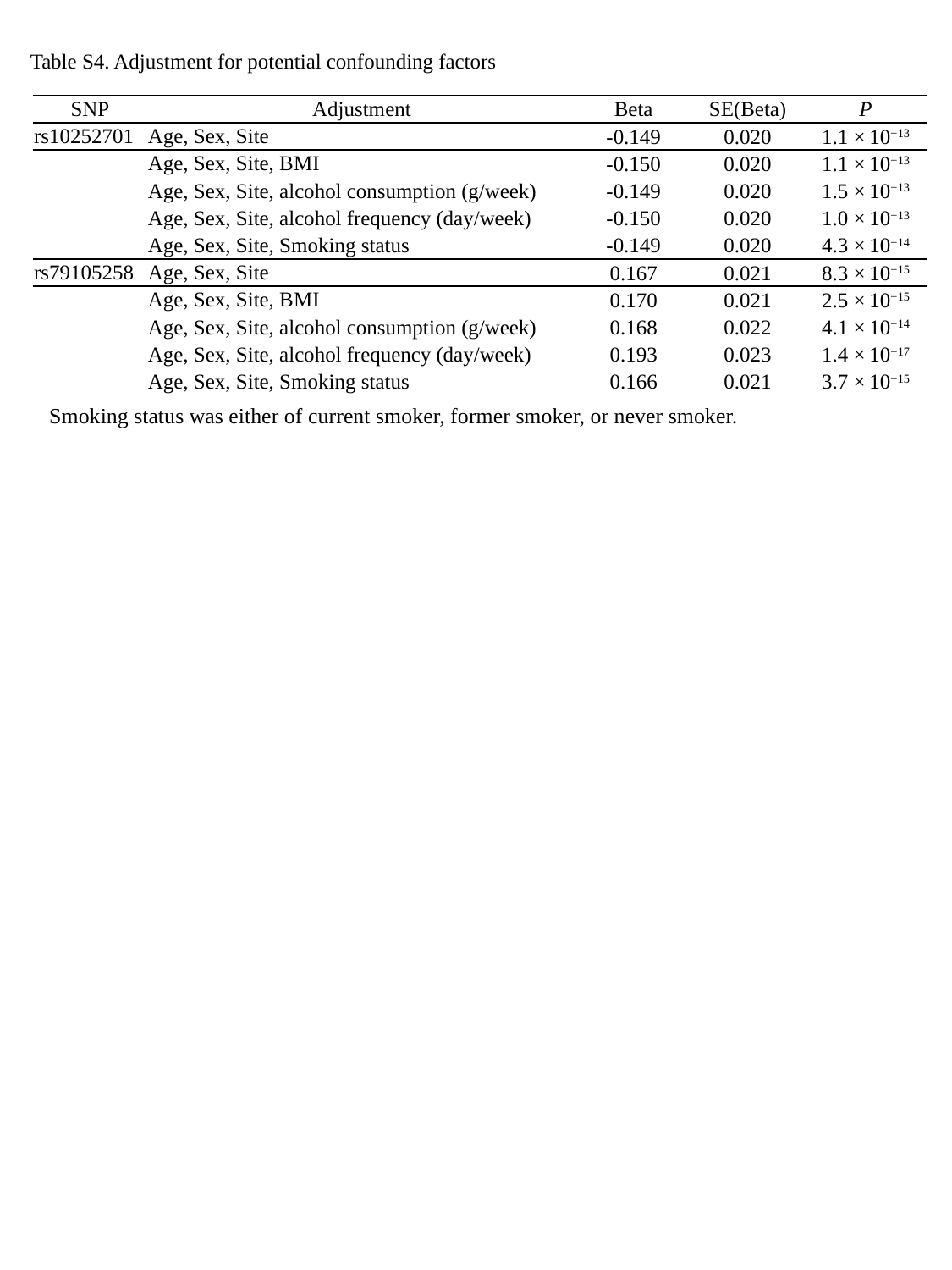

Table S4. Adjustment for potential confounding factors
| SNP | Adjustment | Beta | SE(Beta) | P |
| --- | --- | --- | --- | --- |
| rs10252701 | Age, Sex, Site | -0.149 | 0.020 | 1.1 × 10^−13^ |
| | Age, Sex, Site, BMI | -0.150 | 0.020 | 1.1 × 10^−13^ |
| | Age, Sex, Site, alcohol consumption (g/week) | -0.149 | 0.020 | 1.5 × 10^−13^ |
| | Age, Sex, Site, alcohol frequency (day/week) | -0.150 | 0.020 | 1.0 × 10^−13^ |
| | Age, Sex, Site, Smoking status | -0.149 | 0.020 | 4.3 × 10^−14^ |
| rs79105258 | Age, Sex, Site | 0.167 | 0.021 | 8.3 × 10^−15^ |
| | Age, Sex, Site, BMI | 0.170 | 0.021 | 2.5 × 10^−15^ |
| | Age, Sex, Site, alcohol consumption (g/week) | 0.168 | 0.022 | 4.1 × 10^−14^ |
| | Age, Sex, Site, alcohol frequency (day/week) | 0.193 | 0.023 | 1.4 × 10^−17^ |
| | Age, Sex, Site, Smoking status | 0.166 | 0.021 | 3.7 × 10^−15^ |
Smoking status was either of current smoker, former smoker, or never smoker.

## Slide 5
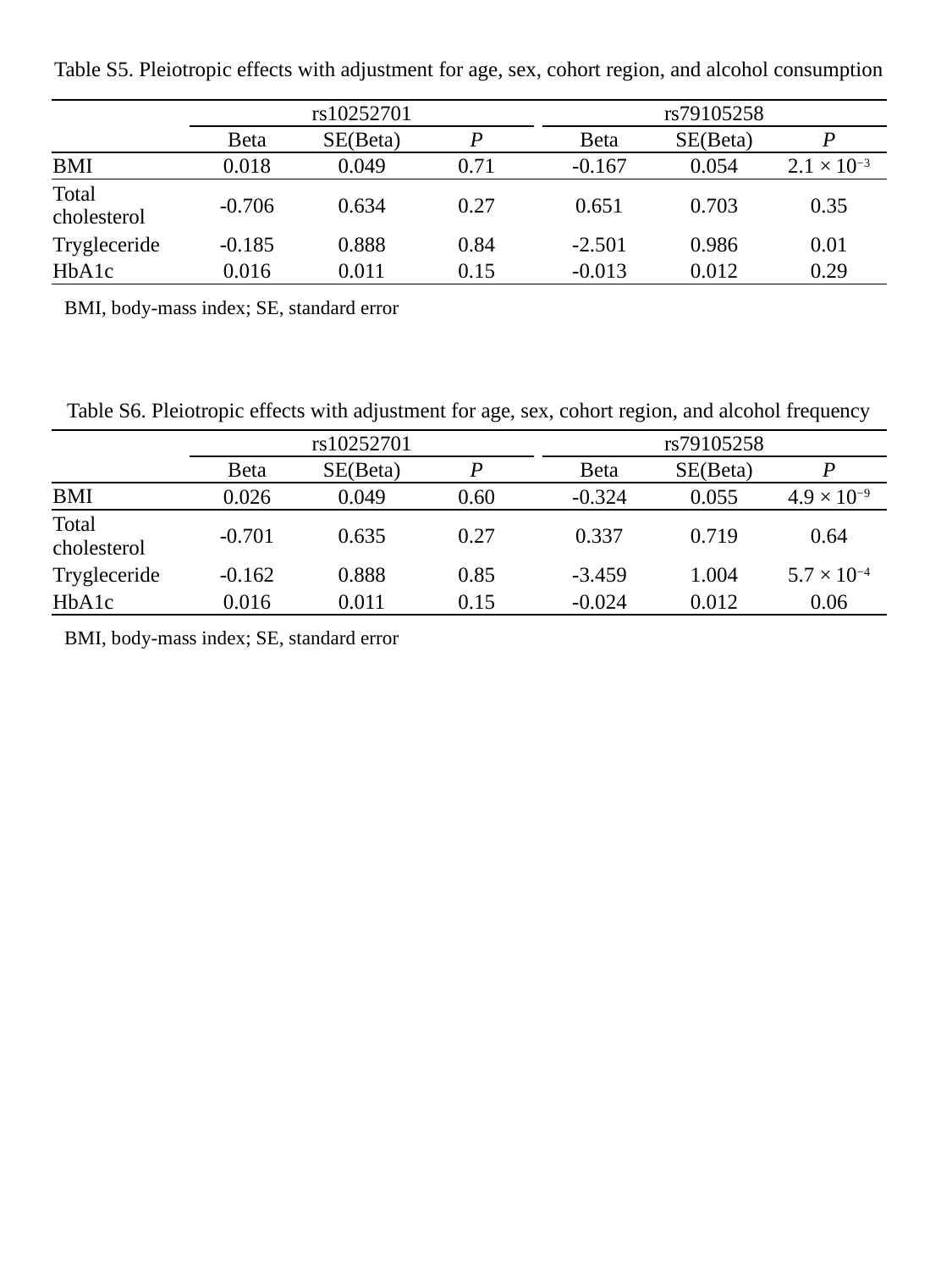

Table S5. Pleiotropic effects with adjustment for age, sex, cohort region, and alcohol consumption
| | rs10252701 | | | | rs79105258 | | |
| --- | --- | --- | --- | --- | --- | --- | --- |
| | Beta | SE(Beta) | P | | Beta | SE(Beta) | P |
| BMI | 0.018 | 0.049 | 0.71 | | -0.167 | 0.054 | 2.1 × 10^−3^ |
| Total cholesterol | -0.706 | 0.634 | 0.27 | | 0.651 | 0.703 | 0.35 |
| Trygleceride | -0.185 | 0.888 | 0.84 | | -2.501 | 0.986 | 0.01 |
| HbA1c | 0.016 | 0.011 | 0.15 | | -0.013 | 0.012 | 0.29 |
BMI, body-mass index; SE, standard error
Table S6. Pleiotropic effects with adjustment for age, sex, cohort region, and alcohol frequency
| | rs10252701 | | | | rs79105258 | | |
| --- | --- | --- | --- | --- | --- | --- | --- |
| | Beta | SE(Beta) | P | | Beta | SE(Beta) | P |
| BMI | 0.026 | 0.049 | 0.60 | | -0.324 | 0.055 | 4.9 × 10^−9^ |
| Total cholesterol | -0.701 | 0.635 | 0.27 | | 0.337 | 0.719 | 0.64 |
| Trygleceride | -0.162 | 0.888 | 0.85 | | -3.459 | 1.004 | 5.7 × 10^−4^ |
| HbA1c | 0.016 | 0.011 | 0.15 | | -0.024 | 0.012 | 0.06 |
BMI, body-mass index; SE, standard error
