# Supplementary material for: GWAS of habitual coffee consumption reveals a sex difference in the genetic effect of the 12q24 locus in the Japanese population
Source: BMC Genet. 2019 Jul 26;20:61. doi: 10.1186/s12863-019-0763-7 (PMC6659273; doi:10.1186/s12863-019-0763-7)
Supplement: Supplementary file 2 — Figure S1. Quantile-quantile plot for the genome-wide analysis of coffee consumption. Figure S2. The genomic regional plot from the association analysis of coffee consumption. Figure S3. Effects of a 7p21 variant on habitual coffee consumption stratified by sex and age. Figure S4. Genetic effect of the 12q24 locus in females before and after menopause. (PPTX 748 kb) [file 12863_2019_763_MOESM2_ESM.pptx]

## Slide 1
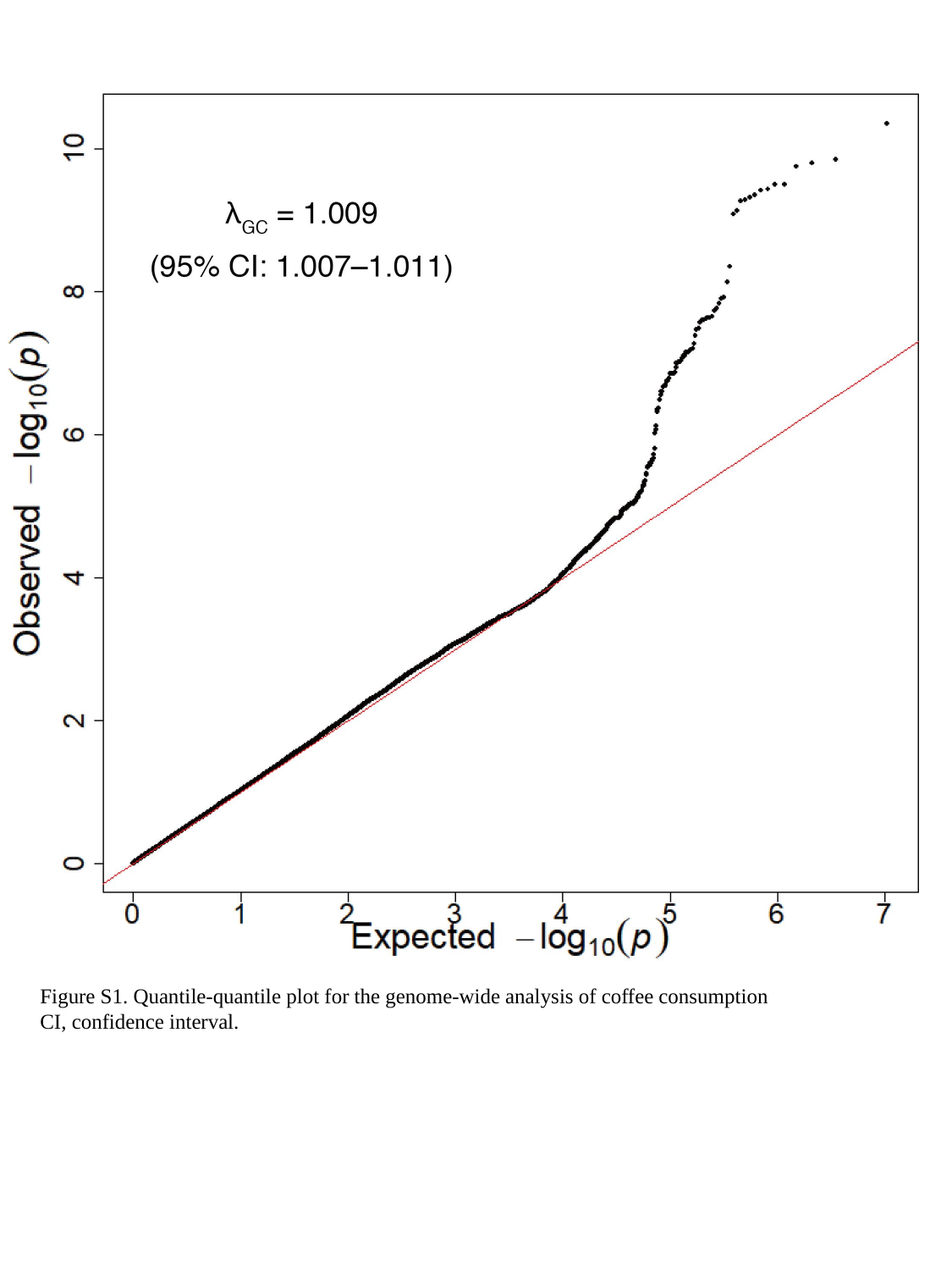

Figure S1. Quantile-quantile plot for the genome-wide analysis of coffee consumption
CI, confidence interval.

## Slide 2
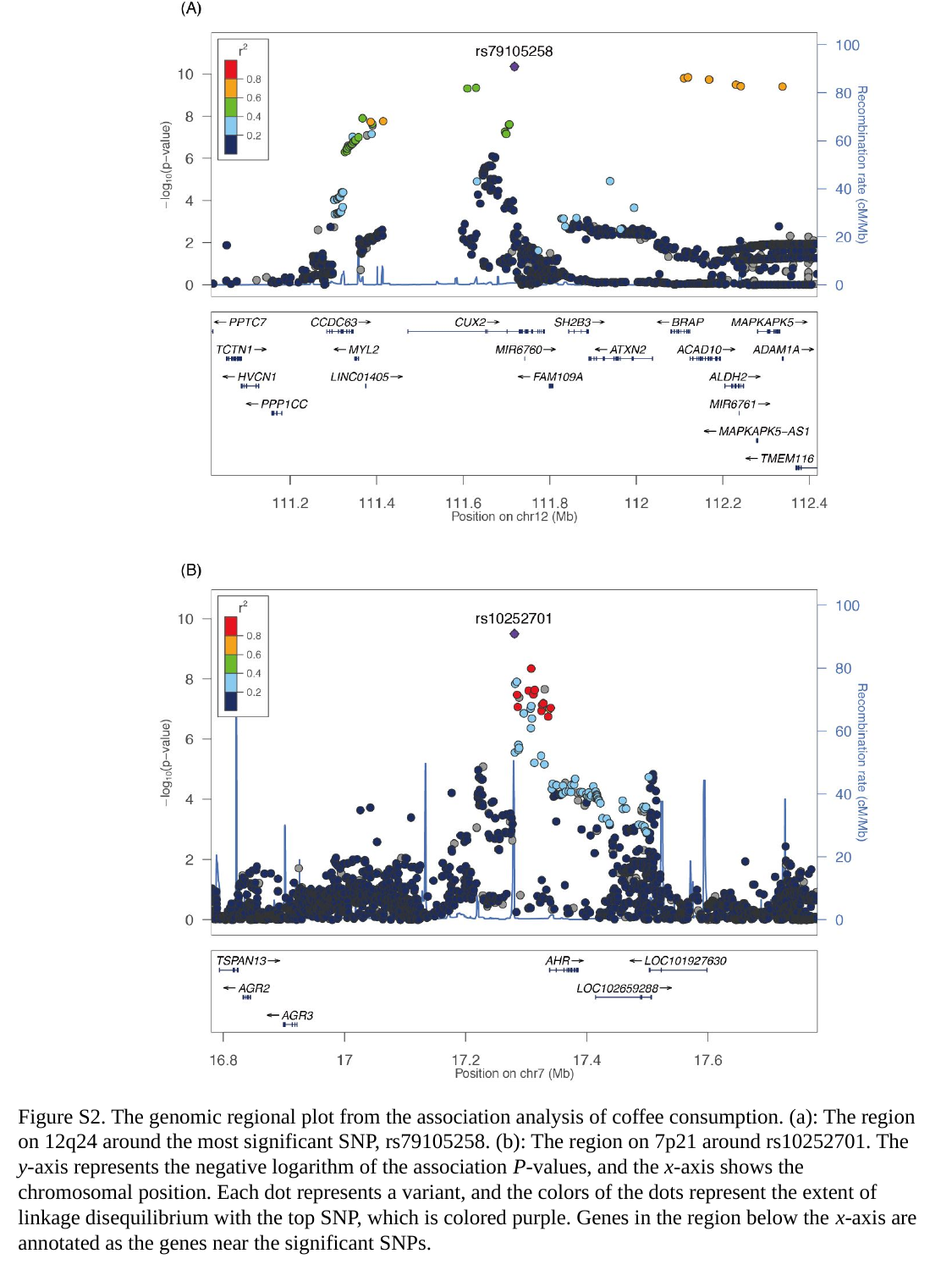

Figure S2. The genomic regional plot from the association analysis of coffee consumption. (a): The region on 12q24 around the most significant SNP, rs79105258. (b): The region on 7p21 around rs10252701. The y-axis represents the negative logarithm of the association P-values, and the x-axis shows the chromosomal position. Each dot represents a variant, and the colors of the dots represent the extent of linkage disequilibrium with the top SNP, which is colored purple. Genes in the region below the x-axis are annotated as the genes near the significant SNPs.

## Slide 3
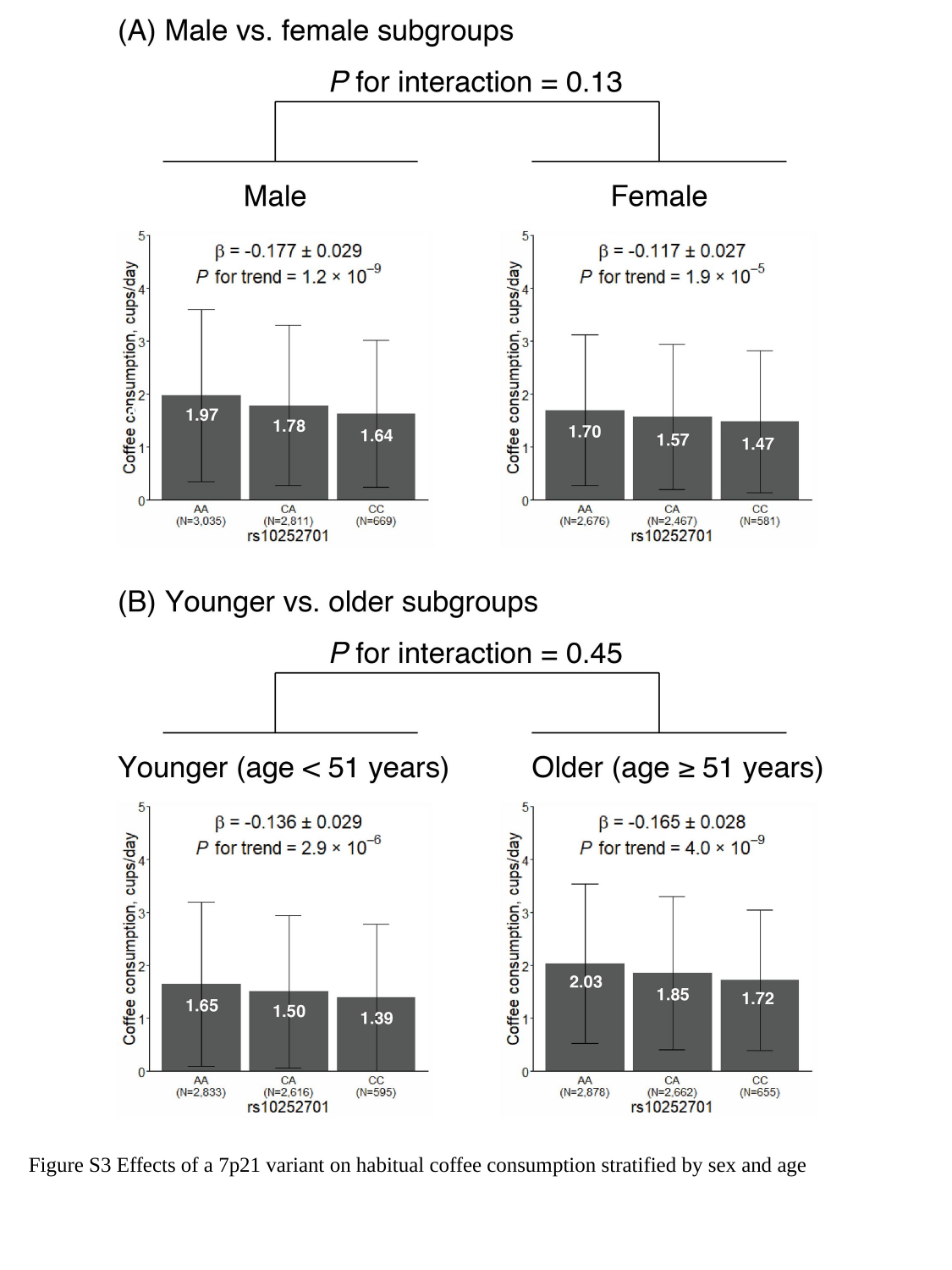

Figure S3 Effects of a 7p21 variant on habitual coffee consumption stratified by sex and age

## Slide 4
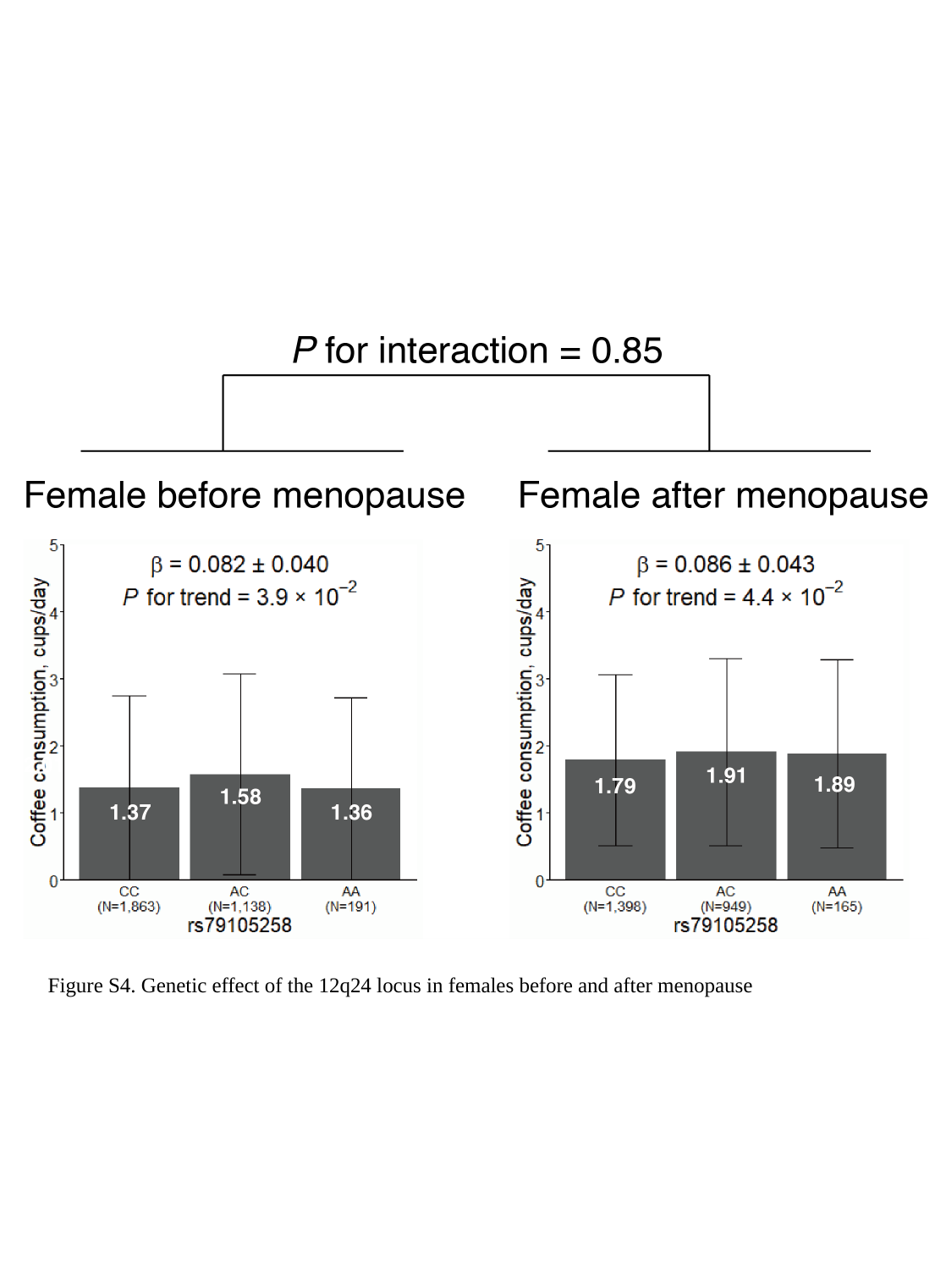

Figure S4. Genetic effect of the 12q24 locus in females before and after menopause
